# Alternative promoter usage of the membrane glycoprotein CD36

**DOI:** 10.1186/1471-2199-7-8

**Published:** 2006-03-03

**Authors:** Malin Andersen, Boris Lenhard, Carl Whatling, Per Eriksson, Jacob Odeberg

**Affiliations:** 1 Department of Biotechnology, AlbaNova University Center, Royal Institute of Technology (KTH), 106 91 Stockholm, Sweden; 2 Bergen Center for Computational Science, Computational Biology Unit, Høyteknologisenteret, Thormøhlensgate 55, N-5008 Bergen, Norway; 3 Atherosclerosis Research Unit, King Gustaf V Research Institute, Karolinska Institutet, Stockholm, Sweden; 4 Department of Molecular Pharmacology, AstraZeneca R&D Mölndal, Sweden

## Abstract

**Background:**

CD36 is a membrane glycoprotein involved in a variety of cellular processes such as lipid transport, immune regulation, hemostasis, adhesion, angiogenesis and atherosclerosis. It is expressed in many tissues and cell types, with a tissue specific expression pattern that is a result of a complex regulation for which the molecular mechanisms are not yet fully understood. There are several alternative mRNA isoforms described for the gene. We have investigated the expression patterns of five alternative first exons of the *CD36 *gene in several human tissues and cell types, to better understand the molecular details behind its regulation.

**Results:**

We have identified one novel alternative first exon of the *CD36 *gene, and confirmed the expression of four previously known alternative first exons of the gene. The alternative transcripts are all expressed in more than one human tissue and their expression patterns vary highly in skeletal muscle, heart, liver, adipose tissue, placenta, spinal cord, cerebrum and monocytes. All alternative first exons are upregulated in THP-1 macrophages in response to oxidized low density lipoproteins. The alternative promoters lack TATA-boxes and CpG islands. The upstream region of exon 1b contains several features common for house keeping gene and monocyte specific gene promoters.

**Conclusion:**

Tissue-specific expression patterns of the alternative first exons of *CD36 *suggest that the alternative first exons of the gene are regulated individually and tissue specifically. At the same time, the fact that all first exons are upregulated in THP-1 macrophages in response to oxidized low density lipoproteins may suggest that the alternative first exons are coregulated in this cell type and environmental condition. The molecular mechanisms regulating CD36 thus appear to be unusually complex, which might reflect the multifunctional role of the gene in different tissues and cellular conditions.

## Background

CD36 is an 88 kd glycoprotein expressed on the surface of many cell types including adipocytes, skeletal muscle cells, platelets, endothelial cells, monocytes and macrophages. It is a membrane protein with a broad ligand-binding specificity and has been postulated to have a variety of functions in lipid transport, immune regulation, hemostasis, signal transduction, adhesion, angiogenesis and atherosclerosis (reviewed in [1-3]). The protein facilitates the membrane transport of long chain fatty acids into muscle and adipose tissue, and CD36 deficiency is associated with a large defect in fatty acid uptake [4]. CD36 is suggested to be involved in the metabolic pathways of insulin resistance [5,6], and it has a major role in the uptake of modified lipoproteins in macrophage foam cells found in atherosclerotic plaques [7].

The tissue specific expression pattern of CD36 is maintained by complex regulatory mechanisms whose molecular details are poorly understood. Interestingly, in tissues central for the energy balance and metabolism (liver, muscle and adipose tissue), the gene has been shown to be regulated tissue specifically in response to specific stimuli such as peroxisome proliferator-activated receptor-γ (PPAR-γ) and retinoid × receptor (RXR) ligands [8]. In diabetic rats, the thiazolidinedione Rosiglitazone significantly activates CD36 expression in adipose tissue and skeletal muscle but not in liver, while the rexinoid LG1002168 activates CD36 in liver and skeletal muscle but not in adipose tissue [8]. Moreover, Type II CD36 deficiency indicates a strong tissue specific control of the gene since the expression is lost on the surface of platelets of affected patients but expressed intact in other tissues [9,10].

Here we have investigated the expression profiles in different tissues and cell types of five alternative first exons of the *CD36 *gene, one of which has not been presented before, with the aim to characterize the alternative promoter usage of the gene and to better understand the mechanisms behind its regulation. We have also performed an *in silico *characterization of the core promoters of the alternative first exons.

## Results

### In silico identification of alternative first exons of *cd36*

Upon inspection of the upstream region of the human *CD36 *gene relative to mappings of known transcripts using the UCSC genome browser [11], we observed several transcribed sequences with different first exons compared to the first published CD36 mRNA sequence [12]. Alternative first exons have previously been reported for the gene [13-15], but the transcript with accession number GenBank:BG944316 has to our knowledge not been analyzed before. The five alternative first exons included in this study are located in a region that spans from 44.7 to 8.5 kilo bases upstream of the translational start site in exon 3.

**Figure 1:**
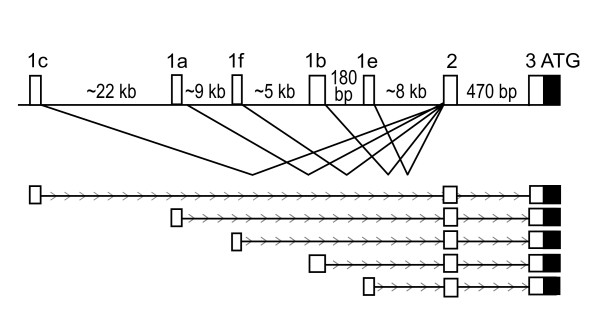
**Overview of the 5'untranslated region of *CD36*. **White boxes represent untranslated mRNA sequence, the black box represent the translated sequence. Exon 1a corresponds to the EST sequence with accession number [GenBank:DA741325]. Exon 1b denotes the alternative first exon with accession number [GenBank:NM_000072]. Exon 1c denotes the alternative first exon with accession number [GenBank:NM_001001547]. Exon 1e denotes the alternative first exon annotated on the sequence with accession number [GenBank:AF266759]. Exon 1f denotes the new alternative first exon identified in this work, corresponding to the EST sequence with accession number [GenBank:BG944316].

The nomenclature used for the alternative first exons of CD36 varies between the publications describing the alternative first exons. For clarity we will follow the nomenclature presented in [15], where exon 1a corresponds to the EST sequence with accession number [GenBank:DA741325], exon 1b corresponds to the RefSeq sequence with accession number [GenBank:NM_000072], exon 1c corresponds to the RefSeq sequence with accession number [GenBank:NM_001001547], exon 1e corresponds to the alternative first exon annotated on the sequence with accession number [GenBank:AF266759] and exon 1f denotes the new alternative first exon identified in this work corresponding to the EST sequence with accession number [GenBank:BG944316] (figure 1).

**Figure 2:**
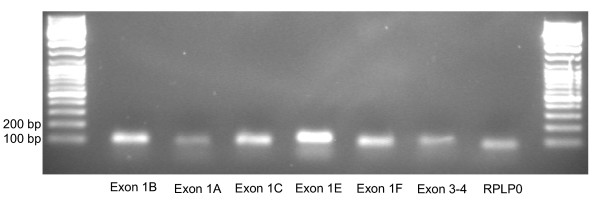
**Agarose gel of PCR products **PCR products from the primers targeted at the alternative first exons of *CD36 *as well as RPLP0, where cDNA from human adipose tissue was used as template and after 45 cycles of PCR. The lanes contain (from left to right) DNA Size ladder (the smallest fragment is 100 bp), RT-PCR products for exon 1b, 1a, 1c, 1e, 1f, 3–4, RPLP0, and finally DNA size ladder.

Primers for real time RT-PCR analysis were successfully designed for the alternative first exons (table 1), and these primers produced specific PCR products of correct sizes (figure 2). The efficiencies of the primer pairs were high (above 85%) and comparable for all primer pairs.

### Expression levels of the alternative first exons in human tissue samples

**Figure 3:**
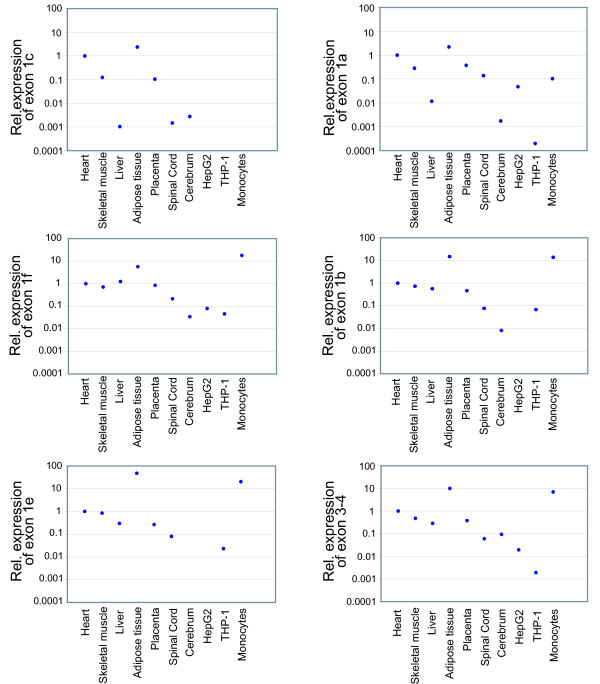
**Relative expression levels of the alternative first exons of *CD36 *in human tissues. **Relative expression levels of the alternative first exons 1a, 1b, 1c, 1e, 1f and exon 3–4 in human heart, skeletal muscle, liver, adipose tissue, placenta, spinal cord, cerebrum, HepG2 cells, THP-1 cells and monocytes. Expression levels of the target exons were normalized to expression levels of RPLP0 and calibrated with the expression level of the corresponding exon in the heart sample.

We investigated the relative transcription levels of the different exons in tissues with a central role in energy metabolism: liver, fat and muscle (heart and skeletal) where the role of CD36 in free fatty acid (FFA) transport is central, as well as in placenta, spinal cord, cerebrum, HepG2 cells, THP-1 cells and monocytes. Exons 1a, 1b, 1c, 1e and the translated part of the gene were most highly expressed in adipose tissue (figure 3). The novel exon 1f was most highly expressed in the monocyte sample, which also expressed relatively high levels of exon 1b, 1e and the translated part of the gene here analyzed. The expression levels of alternative first exons 1a and 1c were relatively high also in heart.

**Figure 4:**
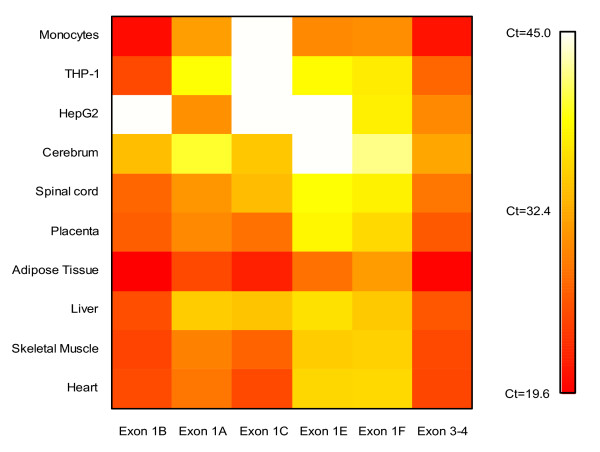
**Alternative first exon usage. **A heat map of the Ct values obtained from RT-PCR analysis of the alternative first exons of CD36 using cDNA corresponding to 70 ng of total RNA from human heart, skeletal muscle, liver, adipose tissue, placenta, spinal cord, cerebrum, HepG2 cells, THP-1 cells and monocytes. Samples are presented in rows and alternative first exons in columns.

A semi-quantitative analysis showed that the expression patterns across the alternative first exons of *CD36 *varied markedly between the tissues (figure 4). The novel exon 1f and the previously described exon 1e were found to be expressed at medium high levels in adipose tissue and monocytes but in low levels in all other tissues analyzed, in a few tissues not even detectable.

### Variation in expression levels of the alternative first exons in monocyte samples from different individuals

**Figure 5:**
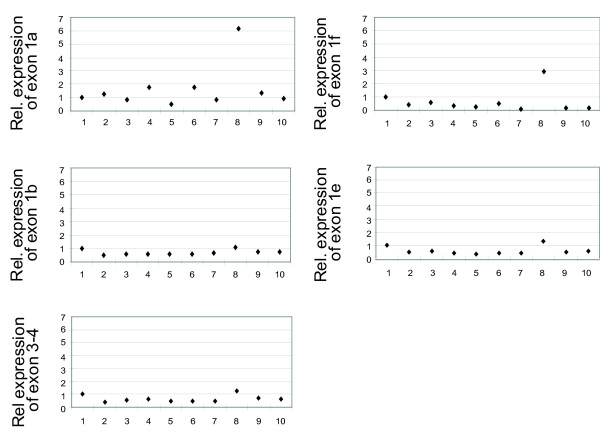
**Relative expression levels of the alternative first exons of *CD36 *in 10 monocyte samples **Relative expression levels of the alternative first exons 1a, 1b, 1e, 1f and exon 3–4 in monocytes samples from 10 individuals. Expression levels of the target exons were normalized to expression levels of RPLP0, and samples were calibrated to the expression level in monocyte sample "1".

We investigated if there were individual variations in expression levels of the *CD36 *mRNA isoforms in different monocyte samples. Quantitative expression analysis performed for one alternative exon at a time using RNA from ten monocyte samples showed that there were considerable differences in expression levels between individuals (except for in exon 1c that was not detected in any of the monocyte samples) (figure 5). Correlation analysis showed that the expression pattern of exon 1b across the human monocyte samples was significantly correlated to the expression pattern of the translated part of the gene (r = 0.78, p < 0.05). The expression patterns of the other alternative first exons were not significantly correlated to the expression pattern of the translated part of the gene.

### Expression of alternative first exons in THP-1 macrophages exposed to oxidized low density lipoprotein

**Figure 6:**
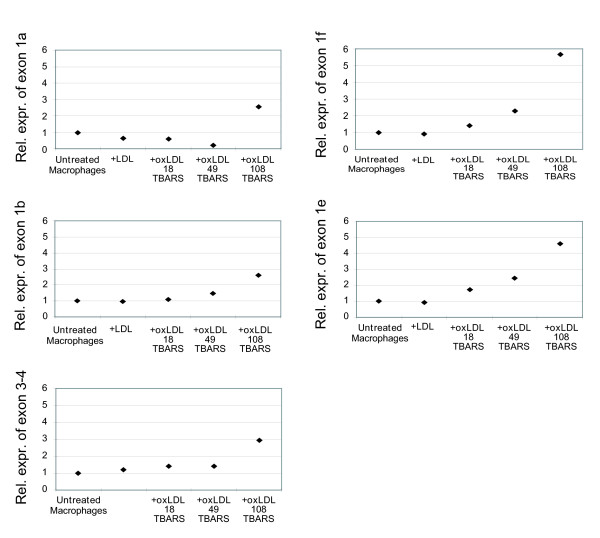
**Relative expression levels of the alternative first exons of *CD36 *in THP-1 before and after treatment with oxidized low density lipoprotein. **Relative expression levels of the alternative first exons 1a, 1b, 1e, 1f and exon 3–4 in normal THP-1 macrophages and in THP-1 macrophages incubated for 24 hours with normal and oxidized LDL of three degrees of oxidation. Expression levels of the target exons were normalized to expression levels of RPLP0, and samples were calibrated to the expression level in the untreated THP-1 cells sample.

It has previously been shown that CD36 is upregulated in cells of monocyte or macrophage lineage after stimulation with oxidized low density lipoproteins (oxLDL) [16,17], but through what regulatory mechanism has not yet been established. To analyze if a subset of the alternative first exons is responsible for this upregulation, we performed expression analysis in THP-1 macrophages (differentiated from monocytes by stimulation with 50 ng/ml PMA for 24 hours) after exposure to oxLDL. THP-1 cells under basal conditions expressed exon 1b most strongly, while exons 1a, 1e and 1f were expressed at lower levels and exon 1c was not detectable (figure 4). After 24 hours exposure of THP-1 cells with a highly oxidized LDL preparation (TBARS 108, see materials and methods), the expression of all the mRNA isoforms present in the cells under basal conditions increased 3-fold to 6-fold (figure 6), which correlates to the 3-fold increase in exon 3–4 expression after the same treatment. The data indicated that all alternative first exons of *CD36 *were close to proportionally upregulated in response to oxLDL, suggesting that a regulatory mechanism in response to oxLDL affects the whole gene locus. Oxidized LDL with TBARS 49 and TBARS 18 appeared to have a small effect, whereas normal LDL had no effect. The same trend in expression patterns was observed also after 6 hours of exposure to the different preparations of oxLDL, although to a lower level. (Data not shown).

### Core promoter structure analysis of the alternative first exons

**Figure 7:**
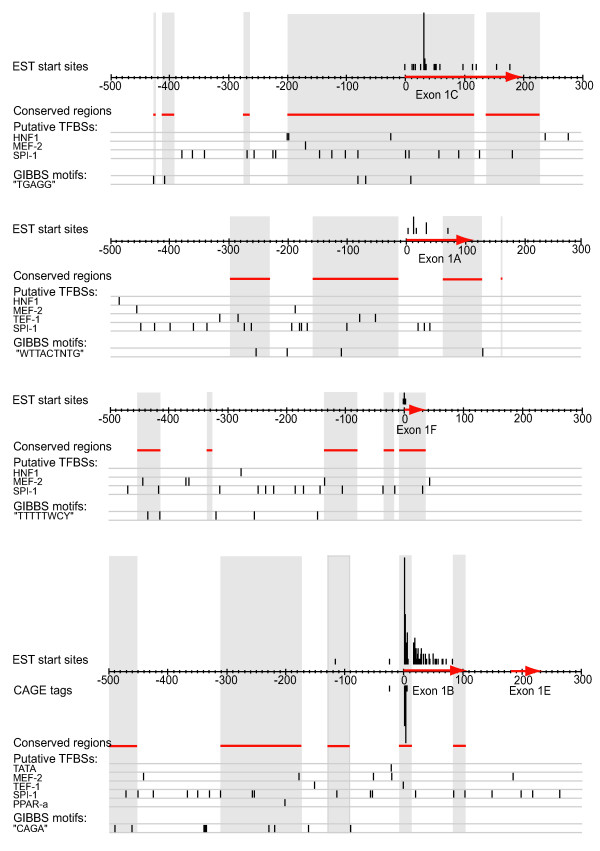
**Summary of the in silico promoter analysis of the alternative first exons of *CD36 ***The x-axes of the figure correspond to the genomic regions from 500 bases upstream to 100 bases downstream of each alternative first exon. Since exon 1e starts only 180 base pairs downstream of exon 1b these two exons are shown on the same genomic sequence. The alternative first exons have been marked out with red arrows on the sequence axes. Tracks from the in silico promoter analysis have been aligned to the sequence axes: Start positions of all GenBank EST sequences have been indicated above the sequences, and the heights of the bars correspond to the number of EST sequences starting at the position. CAGE clusters corresponding to transcription start sites have been marked out below the sequences, and the height of each bar corresponds to the number of CAGE tags in the cluster at the position. Genomic regions conserved in human and mouse have been indicated with red lines below the sequences, and highlighted in grey. Putative binding sites for the transcription factors TATA binding protein, MEF-2, TEF-1, SPI-1, PPAR-α and HNF-1, as well as over-represented motifs detected with Gibbs sampler have been marked out below the sequences.

The result of the promoter analysis is summarized in figure 7. Mapping of start positions of all expressed sequence tag (EST) sequences in GenBank to the genomic sequences corresponding to the alternative first exons showed that exon 1b and 1c have broad promoters with extended transcription start sites (TSS). Although both exon 1b and 1c have one preferred transcription start site each, EST sequence start positions were mapped out both upstream and downstream of the preferred start sites, indicating that the exons may have start sites distributed over up to 100 base pairs each or more. For exon 1b, four clusters of cap-analysis gene expression (CAGE) tags were also present in the CAGE basic viewer provided by FANTOM3 [18,19], coinciding with three of the most common EST sequence start sites extracted from GenBank. For a detailed view of figure 7, with tissue origin of the EST sequences  mapped to the genomic sequence, see Additional file 1. No potential transcription start sites were observed downstream of the PCR primers used in our expression analysis.

Most of the alternative first exons lack a TATA box. There is one TATA box-like sequence at position 28–21 base pair upstream of exon 1b, which is too close to the TSS of the reference cDNA. CpG islands were not detected in or around any of the analyzed sequences.

Multiple instances of putative binding sites for the muscle specific transcription factors MEF-2 and TEF-1, the monocyte and macrophage specific SPI-1, the liver specific transcription factor HNF-1 were observed upstream of the alternative first exons. An evolutionary conserved PPAR-α site was annotated upstream of exon 1b in the UCSC genome browser.

Phylogenetic footprinting against rodent sequences was applied to the sequences to highlight putative transcription factor binding sites in evolutionary conserved sequences. The evolutionary conserved sequences are indicated in figure 7.

Gibbs sampling revealed statistically overrepresented sequence motifs in the alternative promoters. A motif containing the sequence "CAGA" was observed 9 times in front of exon 1b, a motif containing the sequence "WTTACTNTG" was detected 4 times in front of exon 1a, a motif containing the sequence "TGAGG" was detected five times in front of exon 1c and a motif containing the pattern "TTTTTWCY" was observed 4 times upstream of exon 1f. No overrepresented motifs were detected in the sequence between exon 1b and exon 1e, or in the sequence extracted downstream of exon 1e.

## Discussion

Phenotypic complexity of higher eukaryotes is generally believed to be achieved by a more complex regulation of a larger protein repertoire, compared with simpler organisms. The protein diversity in higher organisms results not only from a higher number of genes encoded in the genome, but to a large extent also from alternative splicing of pre-mRNA which generates multiple proteins encoded by the same gene [20]. Transcriptional and regulatory complexity of higher eukaryotes can be further increased by alternative promoters and transcription start sites that are used tissue or developmental specifically, and thus provide means of a complex regulation of the genes [21].

The existence of alternative promoters appears to be a common feature in mammalian genomes. Landry et al estimated that 18% of the genes in the human genome bare evidence of alternative promoter usage [22]. The FANTOM consortium recently concluded from their large data set of transcription initiation and termination sites in mouse that there are 1.3 five prime start sites for each three prime end and 1.83 three prime ends for each five prime start, indicating that the mammalian transcriptome is more complex than previously estimated [19]. As most microarray technologies for analyzing gene expression data only consider one or a subset of all possible splice variants of a gene, information on which alternative first exons contribute to the expression of a gene is often missed in these analyses.

The multifunctional activities of CD36 present in several cell types and tissues suggest that modulation of CD36 expression might lead to a series of potential beneficial or harmful effects in for example atherosclerosis, inflammation, lipid homeostasis, insulin resistance, and angiogenesis, to name a few. However, with few exceptions [14], reports of altered expression of *CD36 *under different conditions in different cell types have been based on analyses targeting the coding regions of the gene, and has not considered alternative transcription start sites. Any attempt to deduce the regulatory mechanisms and pathways behind an observed expression in a cell type or tissue requires knowledge of which alternative first exons and promoters that are involved.

We have identified and confirmed the expression of 5 alternative first exons of *CD36*, all expressed in more than one tissue. Exon 1c was recently described by Noushmehr et al [15], who confirmed its expression in skeletal muscle and islets. Zingg et al [13] described and confirmed the expression of exon 1e in smooth muscle cells and atherosclerotic plaques. Sato et al [14] confirmed the expression of exon 1a in HepG2 cells and exons 1a and 1b in THP-1 cells. According to our data, the levels of expression of the alternative first exons appear to vary between tissues, suggesting that the alternative promoters are regulated tissue specifically.

Expression analysis of the alternative first exons across 10 monocyte samples show that there is a considerable difference in expression levels between individuals and that the expression patterns across the monocyte samples are correlated for exon 1b and the translated part of the gene. This is not surprising since exon 1b is the dominant first exon in monocytes and therefore contributes most to the total CD36 expression in this cell type. The differences in expression levels between individuals may indicate that there are genetic variations between the individuals in the CD36 locus, affecting either regulatory elements of the gene or the stability of the mRNA molecules, perhaps due to untranslated region (UTR) variations.

Alternative promoter usage may not only provide means of an intricate tissue specific regulation of a gene, but could also provide a way of activating a gene in response to various environmental stimuli. It was for example shown by Sato et al [14] that THP-1 cells under basal conditions express the *CD36 *gene both from exon 1b and exon 1a, but only exon 1a was upregulated in the cells in response to PPAR-γ and-α [14]. CD36 is upregulated in THP-1 macrophages also in response to stimulation with oxidized low density lipoprotein (oxLDL) [16]. We studied the expression patterns of the alternative first exons of *CD36 *in THP-1 macrophages after stimulation with oxLDL to analyze if this upregulation occurred through specific alternative first exons. Our results indicate that the activation of *CD36 *transcription in THP-1 macrophages in response to oxLDL affect all alternative first exons proportionally, although the absolute effect is greatest from exon 1b since this exon is most active in THP-1 cells under basal conditions. It is therefore possible that the upregulation of CD36 in response to oxLDL in THP-1 macrophages occurs through a locus control mechanism rather than through a specific regulatory element in one of the alternative promoters.

There are examples in the literature where transcripts from alternative promoters have shown different translational efficiencies, providing post-transcriptional regulation [23]. For CD36 it has been observed that the mRNA transcript starting from exon 1b show increased translational efficiency in response to high glucose levels, primary due to reinitiation of translation after the translation of an upstream untranslated open reading frame (uORF) [24]. We analyzed the 5' untranslated regions of the CD36 transcripts starting with exon 1a, 1c, 1e and 1f, and observed that they all contain at least 2 uORFs upstream of the open reading frame corresponding to the CD36 protein. It can not be excluded that also these transcripts are under a tight translational regulation through similar mechanisms as for exon 1b, either through formation of secondary structures of the mRNA molecules or by uORF usage.

Alternative splicing is described also for internal exons of CD36. Tang et al showed that exon skipping of exons 4 to 5 results in a CD36 isoform lacking amino acid residues 41 to 143 [25]. Kern et al observed a total of 13 alternatively spliced transcripts of the CD36 gene in peripheral blood mononuclear cells (PBMC), and they observed exon skipping of up to 10 out of 12 amino acid coding exons in 8 of the alternative transcripts [26]. Taylor et al described a transcript with an alternative exon 2 (denoted 2b) joined to the common exon 3. Our reverse PCR primers were designed in exon 2a [27], which is found in all but one EST or mRNA sequences in GenBank, while the alternative exon 2b is represented by only one reported mRNA (GenBank: L06850) [27] that lacks exon 1.

The features of the alternative core promoter regions, identified in our in silico analysis of the upstream regions of the alternative first exons, are partly consistent with the expression patterns of the alternative first exons. According to our expression analysis as well as to the EST sequences available in GenBank, exon 1b is expressed in a vide variety of tissues and cell types much like a house keeping gene, and it is highly expressed in monocytes.

The mapping of start positions of EST sequences to the genomic sequence of the alternative first exons of *CD36*, as well as CAGE tag data for exon 1b, indicates that exons 1b and 1c may have extended transcription start sites. It has been observed that housekeeping genes and monocyte and macrophage specific genes often have multiple transcription start sites in a region that can span more than 100 base pairs (Carninci, Sandelin, Lenhard et al, submitted). It cannot be ruled out that the apparent extended transcription start sites of exon 1b and 1c of *CD36 *may reflect artefacts due to incomplete cDNA construction, since library construction information is not available for all of the EST sequences used in our in silico analysis. However, for exon 1b, EST sequences starting upstream of the known 5' site determined with 5'RACE [12] were found, which suggests that the observations can not be all explained by artefacts. The start positions of the EST sequences can however not be assumed to precisely correspond to true transcription start sites. The four clusters of start sites corresponding to exon 1b in the CAGE basic viewer [18] correspond to transcription start sites determined by cap analysis gene expression [28] and confirms that several transcription start sites exist for exon 1b.

Housekeeping genes and monocyte and macrophage specific genes often lack TATA-boxes (Carninci, Sandelin, Lenhard et al., submitted). There is a TATA-box -like sequence in front of exon 1b, but since it is located closer to the major start site of the exon than the optimal spacing of 30–32 base pairs it is unlikely to be functional with respect to that start site. However, the TATA-box like sequence is located 30–32 base pairs upstream of the EST start positions adjacent to the major start site, which means that it might be functional with respect to those start sites.

A PPAR-α binding site was annotated in the upstream region of exon 1b in the UCSC genome browser. This PPAR response element has been shown to be functional *in vitro *for PPAR-γ using super shift and reporter construct analysis [29].

The upstream region of exon 1b contain a high number of SPI-1 sites which are often clustered in the promoter regions of monocyte specific genes, and using Gibbs motif sampler we identified an overrepresented motif containing the element "CAGA", which is another typical promoter element of monocyte specific genes. *In silico *predictions of SPI-1 sites are unfortunately fairly unreliable since the sites are very short and are thus often falsely predicted *in silico*. Several SPI-1 sites were found also in the promoters of the other alternative first exons, although these exons are hardly expressed in monocytes, illustrating this problem. However, it is still possible that the cluster of evolutionary conserved SPI-1 sites and "CAGA" elements can trigger the high expression of exon 1b in monocytes, but without further experimental analyses the significance of these elements remains unknown. Taken together, despite the lack of a CpG island which is often present in the upstream region of a housekeeping gene, the exon 1b promoter has many characteristics of both a house keeping gene promoter and a promoter of a monocyte-specific gene, which is consistent with the expression pattern of this exon.

To further investigate the possibility that the *CD36 *gene might be regulated by a locus-control mechanism that extends to neighbouring genes, we inspected the genomic region in the vicinity of *CD36 *for the presence of genes that are involved in similar biological processes. Some of the genes are expressed predominantly in tissues where CD36 is highly expressed such as muscle (*putative homeodomain transcription factor 2 (PHTF2) *and *calcium channel, voltage-dependent, alpha 2/delta subunit 1(CACNA2D1*)) and heart (*CACNA2D1*). However, most of the genes in the vicinity of *CD36 *are, to our knowledge, not involved in the same biological processes or conditions as CD36, except the gene coding for *hepatocyte growth factor *(*HGF*). A strong association has been observed between serum levels of HGF and the metabolic syndrome [30], although it is unclear if the molecular mechanism for this is related to the function of CD36.

## Conclusion

We have shown that human *CD36 *can be expressed from five alternative first exons, all of which are expressed in more than one tissue. The alternative first exons of *CD36 *appear to be regulated differently in different tissues indicating tissue specific promoters. At the same time, all alternative first exons are upregulated in THP-1 macrophages in response to oxLDL, suggesting that there may be regulatory mechanisms operating on a locus control level in this cell type for this stimulus. This suggests that the molecular mechanisms regulating the *CD36 *gene are unusually complex, which could reflect the multifunctional role of CD36 in different tissues and conditions. In the present study we have established a draft map of how the alternative first exons of *CD36 *are used in a set of human tissues. CD36 is involved in markedly diverse disorders and we anticipate that the presented information will be valuable for further understanding the regulation of the gene, as well as the interpretation of effects of non-coding genetic variation associated with altered expression levels of the gene and disease susceptibility.

## Methods

### Preparation of human monocytes

Peripheral blood mononuclear cells were isolated from buffy coats from healthy donors using Ficoll-Paque Plus (Amersham Pharmacia). The cells were cultured at a density of 5.4 × 106 cells/ml and monocytes were purified by adherence after an overnight incubation of the cells in serum-free medium. Monocytes were then maintained in RPMI 1640 (GIBCO invitrogen cell culture) supplemented with 10% human AB serum (Sigma), 100 U/ml penicillin and 100 μg/ml streptomycin (GIBCO invitrogen cell culture) for 7 days without any change of the medium.

### Cell culture

The human monocyte/macrophage cell line THP-1 was cultured in 10% FCS-RPMI 1640 medium supplemented with 1.0 mM sodium pyruvate and 0.05 mM 2-mercaptoethanol in standard tissue culture flasks in humidified air/CO2 (19:1) at 37°C. Cell density was kept between 0.2 and 1 million cells/ml. To differentiate THP-1 monocytes into THP-1 macrophages, cells were plated at 4 × 105 cells/ml and stimulated with 50 ng/ml PMA for 24 hours [31]. Cells were washed twice with PBS before adding fresh medium and growing for 24 hours.

### Low density lipoprotein (LDL) preparation and oxidation

Human LDL was prepared as described [31]. Briefly, LDL was isolated by sequential density gradient ultracentrifugation using KBr solution (1.030–1.053 g/ml) and dialyzed against PBS. LDL was quantified by measuring ApoB content, using the Bradford assay [31]. Preparations were stored in the dark at 4°C and used within 4 days. For oxidation, LDL (500 μg/ml) were exposed to ultra violet irradiation at 254 nm for a specified time (one to five hours), then sterilized by filtration (0.45μM). Extent of oxidation was determined by measuring the amount of thiobarbituric acid reactive species (TBARS), using malondialdehyde (MDA) standard. TBARS were recorded as nmol MDA per mg LDL protein. LDL, Ox-LDL (50 μg/ml) or PBS carrier was added to THP-1 cells in fresh culture medium 24 hours after the PMA was washed away from the cells, follow by incubation for up to 24 hours

### RNA preparation

RNA from primary monocytes and THP-1 cells was prepared using Trizol according to the manufacturer's instructions and further purified by an RNeasy clean up purification kit (Qiagen). The RNA was finally eluted in RNase free water. RNA concentration was determined spectrophotometrically. In addition, RNA from human tissues was purchased from Stratagene and Clontech. Table 2 contains information of the commercial human tissue RNA samples used in this work. The quality of the RNA was evaluated by gel electrophoresis and by an Agilent 2100 Bioanalyzer (Agilent Technologies) according to the manufacturer's instructions.

### Preparation of cDNA

Total RNA was transcribed into cDNA using the SuperScript III First-Strand Synthesis System for RT-PCR (Invitrogen) according to the manufacturer's instructions, with 200 units of SuperScript III Reverse Transcriptase per reaction, oligo (dT) 20VN primer (manufactured by MWG biotech) and 46°C extension temperature.

### Quantification of expression of alternative first exons

#### Primer design

Primers and TaqMan probes for Real Time RT-PCR analysis of exon 1b, 1a, 1c, 1e and 3 were designed and manufactured by the ABI assays-by-design service (Applied Biosystems). Primers and probe for analysis of exon 1f were designed using the primer express primer design tool (Applied Biosystems) and manufactured by Applied Biosystems. For all targets, a forward PCR primer was designed in each 1st exon with a corresponding reverse primer in exon 2a [27]. TaqMan probes were designed on the boundary between the 1st and 2nd exon, except for the analysis of exon 1f where the probe was designed in exon 2. For the analysis of the transcribed part of the gene, a forward PCR primer was designed in the translated part of exon 3, and the corresponding reverse primer was designed in exon 4. The TaqMan probe was designed on the boundary between the two exons. Primers for the house-keeping gene acidic ribosomal phosphoprotein P0 (*RPLP0*) [32] were manufactured by Thermo Electron. All primer and probe sequences are presented in table 1.

The efficiencies of the primer pairs were obtained by creating dilution series of cDNA and determining the Ct value for each dilution. Ct values versus log cDNA concentration were plotted and the efficiency of each primer pair was calculated using the slope of the curve, according to:

*E *_*primerpair *_= 10^(-1/*slope*) ^- 1[33]

#### Real time RT-PCR

Quantification of the expression of the alternative first exons of CD36 was performed by TaqMan real time semi-quantitative RT-PCR using an ABI PRISM 7000 Sequence Detection System instrument and software (Applied Biosystems). The reactions were performed according to the manufacturer's protocol, using TaqMan Universal Master Mix with UNG, but in 25 μl reaction volumes. All samples were analyzed in triplicates, and at least one negative control was used for each analysis. Standard curves were prepared using dilution series of cDNA prepared as described above.

#### Data analysis

Both the standard curve method and the ΔΔCt method (described by Applied Biosystems in the User Bulletin #2 ABI PRISM 7700 Sequence Detection System [34]) were used to perform quantitative relative expression analysis. Relative expression levels of the transcripts in different samples were compared quantitatively for one alternative first exon at a time and only for samples run in the same PCR setup, to avoid artefacts due to differences in PCR setup or primer efficiency. The expression level of a target transcript was in each sample normalized to the expression level of *RPLP0 *to compensate for differences in RNA loading, and relative expression levels of a transcript in the different samples were obtained by calibrating the normalized expression values of the samples with the normalized expression value of a reference sample. The reference samples were "heart" in figure 3, "monocyte 1" in figure 5, and "untreated macrophages" in figure 6.

In order to make a rough estimation of the relative expression levels of the five alternative first exons in all the analyzed samples, i.e. an inter assay comparison, a heat map of the Ct values obtained from the RT-PCR analysis of the alternative first exons was created, using cDNA corresponding to 70 ng of total RNA for each sample and using the same Ct-threshold on all plates.

The Spearman Rank Correlation of the expression profiles of the alternative first exons in 10 monocyte samples was calculated using the StatView software (SAS), on the relative expression values shown in figure 5 for the alternative first exons and the translated part of the gene. P values of < 0.05 were considered significant.

### In silico characterization of the upstream regions of the alternative first exons

Sequences stretching from 500 base pairs upstream of each alternative first exon to approximately 100 bp downstream of the respective exon were extracted from the UCSC genome browser [11] and used for the in silico promoter analysis. Start positions of all EST sequences from GenBank for *CD36 *were mapped to the extracted sequences. Transcription start site information for the alternative first exons was also extracted from the CAGE basic viewer provided by FANTOM3 [18,19]. The degree of evolutionary conservation around the transcribed region was determined using human and mouse alignments available in the UCSC genome browser [11]. A sliding window of 50 base pairs was used to score every position in the alignment, and positions in the sequence with more than 70 % similarity between the species were considered conserved. Putative TFBSs were mapped out using position specific weight matrix representations of the TFBSs, using the TRANSFAC database of transcription factor matrix profiles and the MatInspector search tool [35] or the JASPAR database [36] with the search tool implemented in Consite [37]. Evolutionary conserved putative transcription factor binding sites were also fetched from the UCSC genome browser [11] and mapped out on the sequences. Gibbs Motif Sampler [38] was used to discover overrepresented sequence patterns within the upstream region of respective promoter.

## Authors' contributions

MA participated in the design of the study, carried out the gene expression analysis, performed the in silico promoter analysis and drafted the manuscript. BL participated in the in silico promoter analysis and helped to draft the manuscript. CW designed and performed the experiments involving oxidation of low density lipoprotein and cultivation of THP-1 cells and revised the manuscript. PE participated in the study design, interpretation of the data, performed the statistical analysis and helped to draft the manuscript. JO conceived the study, participated in the study design, interpretation of the data and helped to draft the manuscript. All authors read and approved the final manuscript.

## Supplementary Material

Additional File 1 **Summary of the *in silico *promoter analysis of the alternative first exons of *CD36*. **The figure shows the genomic sequence stretching from 500 bases upstream of each alternative first exon to approximately 100 bases downstream of each alternative first exon. Exon 1e and exon 1b are shown on the same sequence. Start positions of EST sequences from GenBank are represented by upper case letters in red above the sequence. Different letters represent EST sequences of different tissue origin according to:
H = Heart
L = Liver
A = Adipose
S = Skeletal Muscle
M = Macrophages
E = Erythroid Progenitor Cells
K = Leukopheresis
P = Placenta
C = Umbilical cord
N = Spleen
R = Rectum
I = Ilea Mucosa
T = Thymus
D = Dorsal root ganlion
G = Lacrimal gland
Q = Adrenal gland
B = Whole brain
W = white matter
V = Sympathetic trunk
F = Bone marrow
O = Neuroblastoma
Y = Leiomyosarcoma
J = Jurkat cells
U = Unknown
Sequences corresponding to published exons are underlined, and the coding sequence of the novel alternative first exon 1f is underlined with a dotted line. Putative transcription factor binding sites are underlined with a wavy line, and the name of the corresponding transcription factor is written in blue below the sites. Over-represented motifs detected with gibbs sampler are underlined with a dotted line, and the text "Gibbs motif" is written in blue below the sequence. Click here for file

## Figures and Tables

**Table 1:** Primers and TaqMan probes

Assay	Forward PCR primer	Reverse PCR primer	TaqMan probe
Exon 1c	catctccgaaagcaagctcttcta	aggaaatgaactgatgagtcacagaaa	attggaaagctatcaacttc
Exon 1a	catttgtggccttgtgctctt	tgatgagtcacagaaagaatcaattcgt	atcggacttctaatgatagctt
Exon 1f	ggttacaagcatgacttctattaaacctat	aatgaactgatgagtcacagaaagaatc	tagctttccaatgattagacg
Exon 1b	atgttggagcatttgattgaaaaatcctt	aggaaatgaactgatgagtcacagaaa	attggaaagctatctttaaaatg
Exon 1e	ctgtataaatactcctaagaagttatataggaggacag	caggaaatgaactgatgagtcacaga	cattggaaagctatcttttttc
Exon 3–4	gagacctgcttatccagaagacaat	ttctgtgcctgttttaacccaattttt	aggacaacttgctttt
RPLP0	ccattctatcatcaacgggtacaa	agcaagtgggaaggtgtaatcc	tctccacagacaaggccaggactcgt

**Table 2:** Summary of the commercially available tissues used in this work

Tissue	Supplier	Catalogue number
Adult Skeletal Muscle	Stratagene	Pool of 735029 and 735030
Adult Heart	Stratagene	Pool of 735011 and 735012
Adult Liver, human	Stratagene	Pool of 735017 and 735018
Human Adipose Tissue	Clontech	CR1958
Cerebrum	Clontech	64098-1
Placenta	Clontech	64095-1
Spinal Cord	Clontech	64113-1
