# Supplementary material for: Alternative promoter usage of the membrane glycoprotein CD36
Source: BMC Mol Biol. 2006 Mar 3;7:8. doi: 10.1186/1471-2199-7-8 (PMC1475603; doi:10.1186/1471-2199-7-8)
Supplement: Additional File 1 — Summary of the in silico promoter analysis of the alternative first exons of CD36. The figure shows the genomic sequence stretching from 500 bases upstream of each alternative first exon to approximately 100 bases downstream of each alternative first exon. Exon 1e and exon 1b are shown on the same sequence. Start positions of EST sequences from GenBank are represented by upper case letters in red above the sequence. Different letters represent EST sequences of different tissue origin according to: H = Heart L = Liver A = Adipose S = Skeletal Muscle M = Macrophages E = Erythroid Progenitor Cells K = Leukopheresis P = Placenta C = Umbilical cord N = Spleen R = Rectum I = Ilea Mucosa T = Thymus D = Dorsal root ganlion G = Lacrimal gland Q = Adrenal gland B = Whole brain W = white matter V = Sympathetic trunk F = Bone marrow O = Neuroblastoma Y = Leiomyosarcoma J = Jurkat cells U = Unknown Sequences corresponding to published exons are underlined, and the coding sequence of the novel alternative first exon 1f is underlined with a dotted line. Putative transcription factor binding sites are underlined with a wavy line, and the name of the corresponding transcription factor is written in blue below the sites. Over-represented motifs detected with gibbs sampler are underlined with a dotted line, and the text "Gibbs motif" is written in blue below the sequence. [file 1471-2199-7-8-S1.doc]

**Exon 1C:**

GTGCATGACATTTTCTGCCTCATTAAATGAGGCAAACAAAAAATGACCAAATGACTACTGAAAACATAGAGGAGAAAAAATACTAAGAAAGTTTACAGAGACTTTCCAGTAACAAAAGT

*Gibbs Motif Gibbs Motif SPI-1 SPI-1*

TACTGGAACAAAAATCTGTTTTGTTGACTCAATAGTAATCAATATTCACTTTTATGTTGTTTTATGCTTAAGACAGGAAAACCTGAGCCTTCCTCTTTACTATCAGAGCAAATGGTAAT

*SPI-1 SPI-1 SPI-1*

CATGAAGTGAAGCAAATTGATTATTGATTACTGATTACTTGAATTTCTATGTTTTATAAGTAGAGCAACCATCTGTTAACAGGAATAACAGGTTTTAGCAGGGAAAGGATTAAGGGT

*SPI-1|SPI-1 2 HNF-1 sites MEF-2 SPI-1 SPI-1*

P

TTTATTTTCCTCAACTGAGGCCCAAAATGAGTGAGATTTTTCAAGTTAACTTTCATTCCATTTGGCTCAGGTGTCAGGGATCTATATTTAACTTGTGTTAGGCATGCGTCCGAAGAGCT

*SPI-1 Gibbs Motif MEF-2 SPI-1*

*Gibbs Motif*

H

H

H

H

H

H

H

H

HH

HH H H PH H HHH H P H P

GGAAGGCTGAGGATGTCAATGGCTTTCAGATGTCAGGATAACCTTAAGGATAGATGAAGGGTTGAGAGCCTGTGCCTCATTTCTGAGTTCTCAGCTGCTATGCCGTGGAAATCCTGTTT

*SPI-1 Gibbs Motif SPI-1 SPI-1*

P P

ACTTTCTGCATCTGCTCCTGCAAGACTCTGGAGCCAGTCTTGAGGTCCTACATCTCCGAAAGCAAGCTCTTCTAGAAGTTGGTGAGCAGAATGCTTTTTGTTTAGAAGTGTGGCTAT

*SPI-1 SPI-1*

AAATGTCTTTGTAGTTGATGCACTCTCATCTAGTAAGTAACTATTATTTTTGGGCTCTCTAGAA

*MEF-2 HNF-1*

**Exon 1A:**

TTGCTTAATTCTTTCATATTGCAAAATAAACAGCTATTATAAGCCTGAACTTGTTAGTCTTGCTGGGCCCTGCCCAAGGTTGCCCTCATCTCCAGCTTTCCACAAACTGGAATATTCAC

*HNF-1 MEF-2 SPI-1 SPI-1 SPI-1*

TGATGCTTTGTTTCTTCTCCCAGACCAGGATACATGTTGTTATGTGGTTCCTAGGAGGACTGTAAAGTCCTCTAACAAAGACAAAGGATGGAATGAATCAAGGGAATTACTATGAGAAA

*SPI-1 SPI-1| TEF-1 TEF-1  ^_^*

*SPI-1 Gibbs SPI-1*

*Motif*

*GTAGGGATATCCCTGGAGAGGGGACTGTTTCTGTGTGCTTTTAGACATCACAGAGTAATTGTTTCTTTTTTTAAAGGAAGGAACCAGGGTGAAGAGCAGCTCATTTTTAAGCTGGAGAG*

*Gibbs Motif SPI-1|MEF-2|SPI-1|SPI-1 SPI-1*

GTTAACAAACACTGGCTGCAAGATTTACTTTGGTTTAGGAATACCACTGTCTCACAGGAGTGTCAATCAAAATTCCACAGCCATTCTTCGACACTAGGAGGTGGGACTGACTGATGTAC

*Gibbs Motif SPI-1 TEF-1 TEF-1*

H

H D

U H H U V

AGCAGTGATTTGACCCAGCACTTGGGGCAAACACAACCAGAGTCTTCGGTGTTAATTCCCTGGTCTTCCCAACTAGCATTCTGAAAGTGCATTAACATTTGTGGCCTTGTGCTCTTTCA

*SPI-1 SPI-1 SPI-1*

TCGGACTTCTAATGGTAATTATGCCTGTGTAATCACAGTAAAGTTACCTAATCTAAATATGTAAGATTTCACACTTTGCTTGCAATACTTAAAATTTCCTACTTTGATCTGACT

*Gibbs Motif*

**Exon 1F:**

ATCCAGGGCTATTATAATATTTCTCAGAACTAAAATTTGTGGTACCCTTAATGACAATGAATTTTTTCTTTCTGCCCGGTAAAAATTCAAAATTCATTAATTGCTATTTTTTTCTCAAC

*SPI-1| MEF-2 Gibbs- SPI-1|SPI-1|Gibbs- MEF-2*

*Motif Motif*

GTTTAAAAAACTAGGTGCTCATCAACTAAATGCTTGTTTTGGCTATGAATTATTGATTTTTTTCCTGCTATTTCATCACACCATGCTTTGAGACCTAGTTATTCTTTTGCCTAAATGTT

*MEF-2  ^_ _^  HNF-1*

*Gibbs SPI-1*

*Motif*

TCTGTTTTTTCCTCGAATAATTTCCTATCTACTCTATGAACACAGTTTACCTAACTATGCTGATCATGGCATTTCCTTATGTTTCTTACCATCATTTAAATGTTGACTAATTTTTTTTC

*------------ SPI-1 SPI-1 SPI-1 SPI-1  ^_^SPI-1*

*Gibbs-|SPI-1 Gibbs-*

*Motif Motif*

CTACTTAGAGAATTGTTTTTGCAAGCTGTTCAAGTAAGAATTAGGTCTGCCAACATTTTTAGATGGCCTGTAAAATTTACATAATGATTTGGCCATGCCCACATGAGAACTACCTACAT

*MEF-2 SPI-1 SPI-1*

H

EF E

ATACAGAAGTGCTCTCTTTAATCTGTTACAAGCATGACTTCTATTAAACCTATAGGTAAGATTATTTTCTACTTTTAATGCAGTATGTTTGTTTTTATCCTGACTTTTGATTCATTAGG

*SPI-1 SPI-1 MEF-2*

CCATGTGGTTTAAGTCTAAACTAAGTTACTCTGATA

**Exon 1B och 1E:**

AGTCTCCAATTGTTTTCAGAATTATTAGTCTCACAGTATTTCAGAATTACGCTGTATAAAAAAGTACATTAAATTTAAACTTAATAAAATAAGTTTCGCAAGCTCAGTCAAGACAGGGA

*Gibbs motif Gibbs motif MEF-2 SPI-1 SPI-1*

AGTGAGAATATGATTATTCTTCCAAATTTACTATTTCCACCAGCGGTCTCAGTTCAGCATCTGAGAATATGTTAGGGGAAACTCAGCAAGTCAGTTCCTTTTGTTTTTCTGGCCTCTGA

*SPI-1 3 overlapping Gibbs motifs SPI-1 SPI-1*

CTTACTTGGATGGGAAATAGCCAAAAAAAAAAAAAATGCTGAATATCTCAGATATAGGTAATGGGTCTTCACCAGAACATAAAAATAGACCCTTATTAGCCATATCAGTAATGTGCTGT

*SPI-1 SPI-1 Gibbs- Gibbs- PPAR-α |SPI-1|SPI-1| MEF-2 Gibbs- TEF-1*

*motif motif motif*

P H

GTGGGGGATTTTTTTTTTCTTTCAATTCCTCTGGCAACAAACCACACACTGGGATCTGACACTGTAGAGTGCTTTCTCTTCTCTTTTTTTGGGGGGGGGAGGGGGTGTGGTTGCATATT

*SPI-1 Gibbs motif SPI-1|SPI-1| MEF-2 TATA-*

E

L

K

K

K

K

K

K

U

U

U

UU

UN

UR

UM H

UM O A

UHCK J A

UHHK D MAV H K

UHUK P DPW AI AP H H H P

UHUK H BDPPWWUUQ AAP A H P YP P A G S AA A H

TAAACTCTCACGCATTTATGTACTGAGGACTGCAGTGTAGGACTTTCCTGCAGAATACCATTTGATCCTATTAAGAATTGTCCAAATGTTGGAGCATTTGATTGAAAAATCCTTCTTAG

*Box  ^_^  SPI-1 SPI-1*

*MEF-2 TEF-1*

CCATTTTAAAGGTAAGTTGTATGATTTTTCTTTAAATAAAAAAGATTAAGGGATTTTTCCCATCACCCAGATGACGGGAGGTGACACTACTGTATAAATACTCCTAAGAAGTTATATAG

*SPI-1 MEF-2 SPI-1 MEF-2 SPI-1*

GAGGACAGGAAAAAAGGTAGTAACTGTTTTCTTAGTCACTAGGCTTTATCTAAAAATCAGGTTTGCATT

*SPI-1 MEF-2*
